# Identification of the Bacterial Pathogens in Children with Otitis Media: A Study in the Northwestern Portuguese District of Braga

**DOI:** 10.3390/microorganisms10010054

**Published:** 2021-12-27

**Authors:** Maria Daniela Silva, António Lima, Nuno Marçal, Luís Dias, Miguel Gama, Sanna Sillankorva

**Affiliations:** 1 CEB—Centre of Biological Engineering, LIBRO—Laboratório de Investigação em Biofilmes Rosário Oliveira, University of Minho, 4710-057 Braga, Portugal; mdanielasilva@ceb.uminho.pt (M.D.S.); fmgama@deb.uminho.pt (M.G.); 2 INL—International Iberian Nanotechnology Laboratory, Avenida Mestre José Veiga, 4715-330 Braga, Portugal; 3 Department of Otolaryngology, Hospital de Braga, 4710-243 Braga, Portugal; antoniofonteslima24@gmail.com (A.L.); luisdiasorl@gmail.com (L.D.); 4 Department of Otolaryngology, Trofa Saúde Hospital, 4715-196 Braga, Portugal; nunomarcal.orl@gmail.com

**Keywords:** otitis media, bacteria, prevalence, culture, polymerase chain reaction

## Abstract

Understanding the bacterial etiology of otitis media (OM) is important when designing and evaluating the best course of treatment. This study analyzed middle ear fluid (MEF) and nasopharynx (NP) samples collected from 49 children with OM undergoing myringotomy in the northwestern Portuguese district of Braga. A correlation between species in the NP and MEF was observed following pathogen detection by culture and quantitative polymerase chain reaction (qPCR) methods. Bacterial identification using culturing methods showed that *Moraxella catarrhalis* was the most representative in NP and MEF, followed by *Streptococcus pneumoniae*. However, qPCR of MEF showed a higher prevalence (61%) of *Haemophilus influenzae*. *S. pneumoniae* was not the most frequently identified species, but it still remains one of the leading causes of OM in this region despite 93.9% of the children being vaccinated with the pneumococcal conjugate vaccine. Furthermore, 46% of the samples analyzed by qPCR identified more than two bacterial species. *M. catarrhalis* and *S. pneumoniae* were the most frequent combination identified in NP and MEF samples by culturing methods. Additionally, a few NP and MEF samples simultaneously presented the three main otopathogens. These results point out that polymicrobial infections play an important role in OM. Further studies characterizing the serotypes of the strains isolated, their resistance profile, and their biofilm forming ability would help in the development of more targeted strategies against otitis media.

## 1. Introduction

Otitis media (OM) is a significant public health problem in childhood. Middle ear inflammation is the most frequent reason children visit their doctor, take antibiotics, and undergo surgery [1]. Acute otitis media (AOM) and otitis media with effusion (OME) are the two major sub-classifications of OM. In AOM, the presence of middle ear fluid (MEF) is accompanied by otalgia, otorrhea, and fever, among other signs and symptoms of an acute infection. In OME, which can be developed as a sequel to AOM or as a new onset after viral infection, MEF is present in the absence of signs and symptoms of infection, with the most common alteration detected being hearing loss [2]. Recurrent AOM (RAOM) are very common, as well as persistent OME (chronic OME, COME), with myringotomy and the insertion of ventilation tubes often being performed for their treatment [1].

As well as its clinical relevance, OM also has a significant economic impact associated with medical and nonmedical costs. For instance, in Portugal, the mean costs per doctor-diagnosed OM episode are estimated at 334 euros divided into three categories. The highest share is related to direct medical costs (59.6%), including emergency room visits, pediatrician visits, and antibiotic prescriptions. However, there are also direct nonmedical costs (1.5%), and either indirect nonmedical or travel-related costs (38.9%), e.g., due to the loss of productivity at work, absence from a paid job, or loss of leisure time, among others. Thus, the authors estimated an economic burden of 72 million euros per year to the Portuguese society [3]. In the USA, the annual direct costs associated with OM have been estimated at 3–5 billion US dollars, with indirect nonmedical costs further increasing this value. The cost of one episode can vary between USD 108 and USD 1330 [4].

The nasopharynx (NP) is a key element in the pathogenesis of otitis media, being a reservoir of bacteria that can cause a middle ear infection. A viral upper respiratory tract infection usually triggers the progression of the bacteria from commensal to pathogenic. Genetically similar bacteria have been isolated both from MEF and NP samples [5,6] and from adenoid tissue and MEF [7], suggesting a correlation between the nasopharynx/adenoids and the middle ear microenvironments. 

The three major bacterial pathogens associated with OM are *Streptococcus pneumoniae*, *Haemophilus influenzae*, and *Moraxella catarrhalis*. According to a systematic review, *S. pneumoniae* is the most predominant in AOM patients, while *H. influenzae* is the most frequently identified in patients with COME, RAOM, and AOM with treatment failure. This result is consistently obtained using microbiological culturing techniques and polymerase chain reaction (PCR) [8]. However, the use of molecular methods with improved sensitivity compared to bacterial culture allows the identification of other bacterial pathogens. For instance, *Alloiococcus otitis* and *Turicella otitidis* have been detected by PCR or 16S rRNA gene sequencing with higher frequency from the MEF samples of OM patients [9,10,11,12,13]. Other bacterial species identified in OM cases with lower prevalence include, for instance, *S. aureus* and *P. aeruginosa* [8,11,14].

Studies on the identification and prevalence of the bacterial species associated with OM are critical to developing and evaluating therapeutic approaches and the impact of vaccination programs [8,15]. Bacterial culture has been the standard method for identifying the AOM bioburden. However, the percentage of culture-positive results decreases in MEF samples from children with RAOM, AOM with treatment failure, and, particularly, OME. The reduced growth of some bacterial species, the presence of inhibitory substances, previous antibiotic therapy, and biofilms are some factors that might affect the detection by culture methods [11,16,17]. PCR improves the sensitivity of pathogens’ detection in samples from middle ear infections, increasing the prevalence of positive culture findings and allowing the detection of new bacterial species, as described above [18,19]. 

In Portugal, to our knowledge, only one study evaluating the bacterial etiology of OM has been performed. This study focused on children with AOM with spontaneous otorrhea. Of the 113 samples from ear discharge analyzed, 48.7% were culture positive for bacteria. Among these, *S. pneumoniae* was present in 50.9% (multiple serotypes), *Streptococcus pyogenes* in 30.9%, *M. catarrhalis* in 27.3%, and *H. influenzae* in 20.0%. Furthermore, 25.4% of the samples presented two or more of these species [20]. 

This study aimed to determine the prevalence of otopathogens in children with OM undergoing myringotomy in the northwestern Portuguese district of Braga. MEF samples were studied with conventional culture and quantitative PCR (qPCR) techniques to detect *S. pneumoniae*, *H. influenzae*, *M. catarrhalis*, *S. aureus*, and *P. aeruginosa.* Additionally, bacterial culture of nasopharyngeal swabs was performed and compared with the results obtained with MEF samples from the same patient to establish a possible relationship between them. 

## 2. Materials and Methods

### 2.1. Patients and Sample Collection

This study included children (<12 years of age) with a diagnosis of acute otitis media (AOM) or otitis media with effusion (OME) and indication for myringotomy attending the Public Hospital of Braga between January 2018 and February 2020. Patients with a history of malignancy, organ transplantation, systemic infectious diseases, and immune deficiency were excluded.

Samples from the nasopharynx (NP) and the middle ear fluid (MEF) were collected during the surgery. Patients’ relevant data were registered in a form by the physician responsible for the surgery collecting different data including age, gender, type of otitis media (acute, with effusion, recurrent, chronic), laterality, myringotomy (first intervention or reintervention), antibiotics prescribed prior to surgery, PCV vaccination status. NP samples were collected by gently rotating a sterile cotton wool swab in the nasopharynx, then placed on Amies transport media (Deltalab 300284 or VWR 710-0438P). Before the surgical procedure, the external ear was disinfected with povidone-iodine for 2 min, then a washing step with saline (NaCl 0.9%) was performed to eliminate the antiseptic agent. After myringotomy, MEF was aspirated using a suction device coupled to a collector (Vygon 5534.06). The ethical committee of the hospitals approved the study, and written consent was obtained from all patients involved.

### 2.2. Bacterial Culture

Samples were stored at 4 °C temperature and transported to the laboratory. Once at the lab, NP swabs were immediately cultured (as described below). At the same time, MEF samples were divided into two fractions: one was immediately used for bacterial culture, and another was stored at −80 °C for subsequent bacterial DNA extraction.

All NP swabs and MEF specimens were examined for specific bacterial species using conventional culture methods. Briefly, samples were streaked on 5% (*v*/*v*) sheep blood agar and chocolate agar and incubated at 37 °C with 5% CO_2_ for 24 to 48 h. They were also streaked on 5% (*v*/*v*) sheep blood agar and MacConkey agar and incubated at 37 °C, aerobically, for the same period. 

Colonies were inoculated on the following selective media for identification of: (i) *S. pneumoniae*—sheep blood agar (Tryptic Soy Broth (TSB) + 1.2% (*w*/*v*) agar with 5% sheep blood) supplemented with colistin (5 µg/mL) and oxolinic acid (2.5 µg/L); (ii) *H. influenzae*—chocolate agar (TSB + 1.2% (*w*/*v*) agar with 5% horse blood heated at 55 °C) supplemented with vancomycin (5 µg/mL), clindamycin (1 ug/mL), and bacitracin (300 µg/mL); (iii) *M. catarrhalis*—chocolate agar with vancomycin (5 µg/mL), trimethoprim (3 µg/mL), amphotericin B (2 µg/mL) and acetazolamide (10 µg/mL); (iv) *Staphylococcus aureus*—mannitol salt agar (MSA); (v) *Pseudomonas aeruginosa*—*Pseudomonas* isolation agar (PIA).

Species identification was also based on specific tests, such as catalase and oxidase tests. *S. pneumoniae* was specifically identified by the optochin disk test and *H. influenzae* by the V and X factor growth requirements.

### 2.3. Specific Quantitative PCR of MEF Samples

Detection of specific individual bacterial pathogens in MEF samples was also evaluated by quantitative PCR.

Calibration curves were performed using DNA extracted from individual strains. Briefly, DNA was extracted from cultures of *S. pneumoniae* R6st, *H. influenzae* C894248, *M. catarrhalis* U225012, *S. aureus* ATCC 6358, and *P. aeruginosa* PAO1 (Table 1) using the E.Z.N.A. Bacterial DNA Kit (OMEGA bio-tek) following the manufacturer’s instructions. The concentrations and purity ratios were measured (Nanodrop), and the DNA integrity was confirmed by agarose gel electrophoresis (2% agarose gel). Initially, primers described by Sillanpaa et al. (2016) were tested by conventional PCR. Amplification was performed using primers (Table 2) [11]. Three pairs of primers targeting the 16S rRNA genes of *S. pneumoniae*, *H. influenzae*, and *M. catarrhalis*, originally reported by Hendolin et al. (1997), were also used [17]. The multiplex-PCR reaction described by the authors was adapted for KAPA-Taq DNA Polymerase conditions. The other three pairs of primers target the *lytA* gene of *S. pneumoniae*, the *nucA* gene of *S. aureus*, and the *oprL* gene of *P. aeruginosa*. Genes were amplified by PCR using KAPA-Taq DNA Polymerase following the manufacturer’s instructions, and amplification confirmed by agarose gel electrophoresis (Figure S1). Then, calibration curves by quantitative PCR were performed using 1:10 DNA dilutions for each bacterial species and Xpert Fast SYBR Mastermix (Grisp, Portugal) following the manufacturer’s instructions (Figures S2 and S3).

MEF samples (approximately 250 µL) were defrosted and spiked with 1 µL of a plasmid (empty pET-28a(+)) as an exogenous internal control. The DNA was extracted using the E.Z.N.A. Universal Pathogen Kit (OMEGA bio-tek) following the manufacturer’s instructions. The detection of the specific pathogens was performed by individual qPCR reactions using the primers described above and Xpert Fast SYBR Mastermix (Grisp, Portugal), in duplicate. The exogenous internal control was detected using the universal T7 primers (Forward (5′ → 3′): TAATACGACTCACTATAGGG; Reverse (5′ → 3′): GCTAGTTATTGCTCAGCGG).

## 3. Results

### 3.1. Study Population

**Figure 1:**
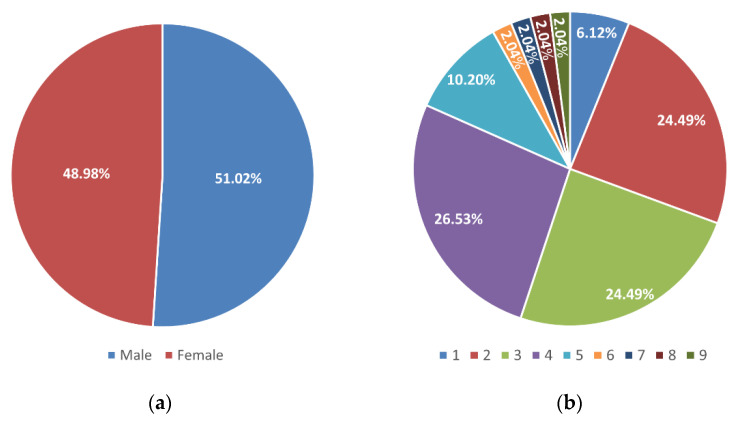
Distribution of samples based on patients’ sex (**a**) and age (**b**).

From January 2018 to February 2020, 49 samples were collected from the nasopharynx (NP) and the middle ear fluid (MEF) of children with OM. The patients’ sex was well-distributed, and the mean age of the individuals was 3.5 years (±1.7), ranging from 1 to 9 years old, and distributed as in Figure 1. 

Patients’ history questionnaires were filled in by the responsible doctors and are compiled in Table S1. Seven of the cases were classified as RAOM, while the other 42 were due to OME, of which 15 were COME. Moreover, 29 children had bilateral OM, while the remaining 20 had unilateral OM on the right (15) or left (5) ear. The myringotomy was performed for the first time, except in two cases where these were reinterventions (Table S1, patients 1 and 2). Furthermore, in only two cases, adenoidectomy was not performed at the same time as the myringotomy. Only three of these children had not been vaccinated with a pneumococcal conjugate vaccine (PCV). Moreover, 57% of the children had not taken antibiotics in the last six months. Most of the children taking antibiotics one week to six months before the myringotomy received amoxicillin/clavulanic acid, while a few received penicillin (2), ceftriaxone (1), or cefixime (1) six months prior to the surgery. 

### 3.2. Identification of Bacterial Species

The presence of bacterial pathogens in NP and MEF of 49 children with OM was detected by culture, and MEF samples were further evaluated by qPCR.

**Figure 2:**
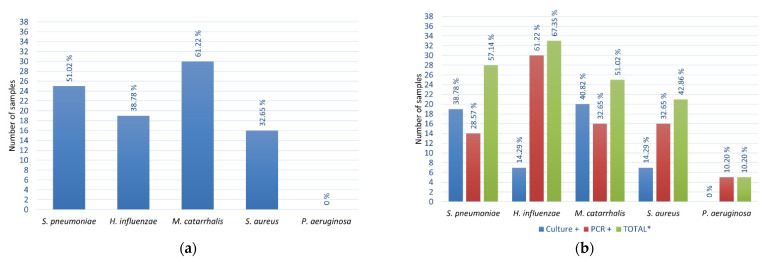
Number of bacterial isolates (percentage of total patients/samples) identified by (**a**) culture in nasopharyngeal samples and (**b**) culture and qPCR in middle ear fluid samples from children with OM. * The total corresponds to the sum of Culture+ and PCR− with Culture− and PCR+ and Culture+ and PCR+ samples.

**Figure 3:**
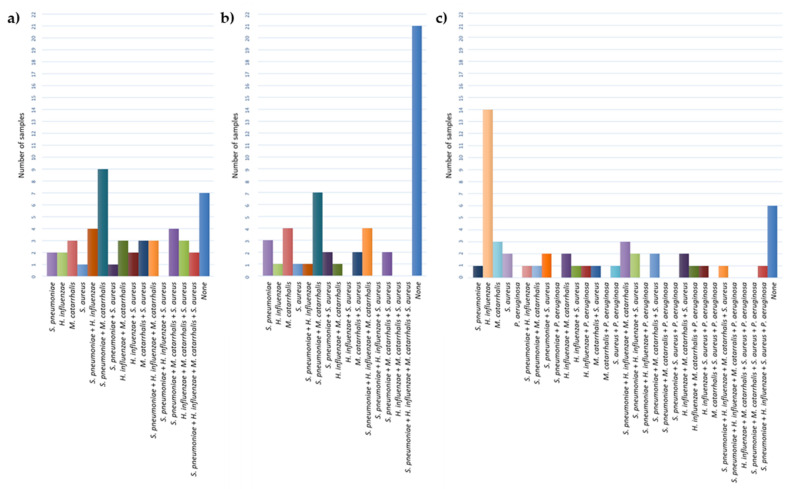
Species combinations in NP and MEF: (**a**) Species in NP identified by culture; (**b**) Species in MEF identified by culture; (**c**) Species in MEF identified by qPCR.

Bacteria were isolated from 85.71% of NP samples using culture-based methods. *M. catarrhalis* was the most commonly isolated species (61.22%), followed by *S. pneumoniae* (51.02%), *H. influenzae* (38.78%), and *S. aureus* (32.65%) (Figure 2a). *P. aeruginosa* was not found in any NP samples. The presence of *M. catarrhalis* was often accompanied by *S. pneumoniae* (18.37%) (Figure 3a). 

Concerning the MEF samples, otopathogens were identified in 93.88% of them, either by culture or qPCR, with 57.14% or 89.80% of the samples being Culture+ or PCR+, respectively, for at least one pathogen. The most common bacteria isolated by culture from the MEF (Figure 2b) was again *M. catarrhalis* (40.82%), followed by *S. pneumoniae* (38.78%), with *H. influenzae* and *S. aureus* being isolated from the same number of samples (14.29%). *M. catarrhalis* was once more frequently found in co-occurrence with *S. pneumoniae* in the MEF (Figure 3b). However, by qPCR methodology, *H. influenzae* was detected in higher numbers (61.22%) (Figure 2b). Both *M. catarrhalis* and *S. aureus* were detected in 32.65% of samples, while *S. pneumoniae* was detected in 28.57% of the individuals. Regarding *P. aeruginosa*, its presence was detected in 10.20% of samples. Overall, combining the two methods (samples Culture+ or PCR+), *H. influenzae* was the most commonly identified pathogen (67.35%), followed by *S. pneumoniae* (57.14%), *M. catarrhalis* (51.02%), *S. aureus* (42.86%), and, lastly, *P. aeruginosa* (10.20%) (Figure 2b). 

*H. influenzae* was detected alone by qPCR in 28.57% of the total MEF samples (Figure 3c). The presence of two or three different species was identified in 20.41% and 22.45% of samples, respectively, while in 4.08% of samples, four pathogens were detected.

**Figure 4:**
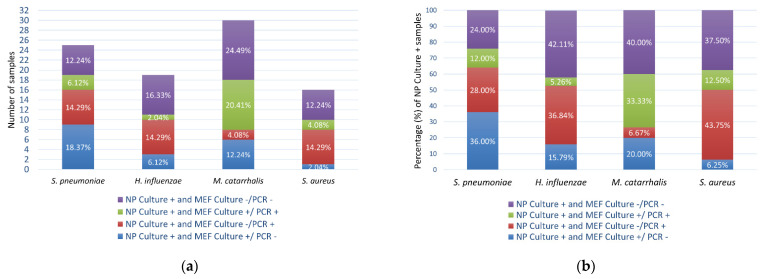
Correspondence between the nasopharynx and the middle ear fluid: (**a**) Number of bacterial isolates (percentage of total patients/samples) detected in the nasopharynx and middle ear fluid of the same patient; (**b**) Percentage of bacterial isolates detected in the middle ear fluid that were isolated from the nasopharynx.

More than half of the NP Culture+ isolates were also detected in the MEF sample of the same patient by culture or qPCR (Figure 4). For instance, 76.00% of the *S. pneumoniae* Culture+ NP samples were positive for *S. pneumoniae* in the MEF sample, of which 48.00% were culture positive (36.00% Culture+/PCR+ plus 12.00% Culture+/PCR+) and 40.00% qPCR positive (28.00% Culture−/PCR+ plus 12.00% Culture+/PCR+) (Figure 4b). 

## 4. Discussion

The prevalence of bacterial pathogens in children with otitis media has been investigated in different European countries [21,22,23,24], but only one study (which analyzed samples from AOM cases with spontaneous otorrhea) has been performed in Portugal [20]. The implementation of the PCV has been reported to impact OM etiology. This vaccine was first licensed in Europe in 2001, and, since then, countries have been introducing it in their national immunization programs. It started as a 7-valent vaccine (PCV-7), protecting against seven pneumococcal serotypes, later substituted by the PCV-10, and then by the PCV-13 [25]. In Portugal, the PCV-13 was introduced in the routine childhood immunization program in 2015, with the different doses being administered at 2, 4, and 12 months of age [26], similarly to most European countries [27]. Before that, it was recommended and free to children at increased risk for pneumococcal disease [28]. The percentage of children immunized in Europe is high (80%) [29], as were those from which samples were collected (93.88%). After the implementation of PCV, studies reported a change in pathogen dominance from *S. pneumoniae* to *H. influenzae* (in France and Greece) [21,22], as well as the replacement of *S. pneumoniae* serotypes covered by the vaccine by others (in France and Spain) [21,24,30]. Moreover, the high use and misuse of antibiotics may have impacted the prevalence of some bacterial species and serotypes [22]. Amoxicillin–clavulanic acid, the gold standard of otitis media treatment, is the antibiotic most commonly used in Portugal, including in the pediatric population [31]. Importantly, 42.86% of the children included in this study had taken amoxicillin-clavulanic acid up to six months before the myringotomy. Despite this, the antibiotic consumption rate in Portugal is below (but near) the average of European countries [31].

In this study, *M. catarrhalis* was the most frequent pathogen isolated by culture from NP samples, being in agreement with studies that report this pathogen as the most common NP colonizer in both children with OM or who are healthy [16,32,33]. *M. catarrhalis* was frequently associated with *S. pneumoniae*. While colonization by *M. catarrhalis* alone is not reported to be associated with an increased risk of OM development, this is significantly enhanced when it co-colonizes the NP with *S. pneumoniae* [33]. The co-infection with *M. catarrhalis* affected the middle ear ascension of *S. pneumoniae* in mice and chinchilla models [34]. Moreover, the co-colonization with *M. catarrhalis* may negatively impact the treatment since β-lactamase-producing *M. catarrhalis* can confer passive protection to *S. pneumoniae*, promoting resistance to the first-line antibiotic amoxicillin [34].

Most of the samples analyzed are from OME cases (85.71%). Therefore, the identification of *H. influenzae* as the most prevalent pathogen in the MEF agrees with other studies that report *H. influenzae* as the predominant pathogen in OME [8,14,35]. However, the reported prevalence rates are highly variable due to different health care programs, particularly vaccination, in various geographical locations [36]. The fact that most children were immunized with a pneumococcal conjugate vaccine possibly contributed to a lower incidence of *S. pneumoniae*. The identification of *S. aureus* in 42.86% of samples may look somewhat surprising, but a recent study also reports a high prevalence (27.78%) of *S. aureus* in MEF samples from OME cases [14]. Nevertheless, differences in pathogens’ prevalence in studies conducted in the same country have been observed. For instance, two studies conducted in Iran reported a prevalence of *H. influenzae* of 95.2% in the city of Shiraz [37] and 11% in the city of Tehran [38] using PCR methods. The diversity of the results available may also result from variability in sample collection, the population studied, specific disease stage, and, of course, the detection techniques used (culture, PCR, next-generation sequencing) [36]. 

PCR methods usually improve the detection of bacteria in MEF samples to a magnitude that varies among the available reports [8,11,39]. Indeed, in the present study, the detection of *H. influenzae* was largely improved using qPCR compared to culturing (Figure 2b) by 4.3 times. This is in agreement with previous studies, where, for instance, increased detection of *H. influenzae* of five [40] or ten [19] times using PCR methods compared to culture was reported. *H. influenzae* is a fastidious organism that is difficult to culture, which could have affected its detection by this approach. Moreover, the fact that PCR methods do not depend on bacterial viability could also contribute to their increased sensitivity [19]. However, it has been shown that the DNA of nonviable bacteria does not persist in the MEF and cannot be detected by PCR 3 days after inoculation in the ME of chinchillas [41]. In contrast, antibiotic-treated bacteria persist in the MEF for weeks and can be detected by PCR but not by culture [41]. Furthermore, the presence of bacterial biofilms has been extensively pointed to being one of the main reasons for Culture−/PCR+ results [42,43].

However, qPCR did not increase the detection of *S. pneumoniae* and *M. catarrhalis*, which might be related to the DNA extraction method. It should be noted that, in some cases, only a few colonies (one or two) were isolated from the MEF samples, and the levels of bacteria in the sample might not have been enough for successful DNA extraction. While this hypothesis could be valid, we used an exogenous internal control, consisting of empty plasmid pET-28a(+), to safeguard against the loss of DNA during the extraction procedure or the presence of PCR inhibitors in the sample. This control was successfully detected in 91.84% of the samples. The nature of the sample, such as the viscosity, or the presence of inhibitors, such as blood, might have interfered in the DNA extraction process and may explain the lack of success in detecting the exogenous internal control in 8.16% of samples.

In 28.57% of the MEF samples, *H. influenzae* was detected alone. However, two different species were present in 20.40% of the samples (4.08% *S. pneumoniae* and *S. aureus*, 4.08% *H. influenzae* and *M. catarrhalis*, 2.04% *S. pneumoniae* and *H. influenzae*, 2.04% *S. pneumoniae* and *M. catarrhalis*, 2.04% *H. influenzae* and *S. aureus*, 2.04% *H. influenzae* and *P. aeruginosa*, 2.04% *M. catarrhalis* and *S. aureus*, 2.04% *S. aureus* and *P. aeruginosa*). Furthermore, 22.45% of samples presented three different bacterial species (6.12% *S. pneumoniae*, *H. influenzae*, and *M. catarrhalis*; 4.08% *S. pneumoniae*, *H. influenzae*, and S. *aureus*; 4.08% *S. pneumoniae*, *M. catarrhalis*, and *S. aureus*; 4.08% *H. influenzae*, *M. catarrhalis*, and *S. aureus*; 2.04% *H. influenzae*, *M. catarrhalis*, and *P. aeruginosa*; 2.04% *H. influenzae*, *S. aureus*, and *P. aeruginosa*). In 4.08% of samples, four pathogens were detected (2.04% *S. pneumoniae*, *H. influenzae*, *M. catarrhalis*, and *S. aureus*; 2.04% *S. pneumoniae*, *H. influenzae*, *S. aureus*, and *P. aeruginosa*). These show that polymicrobial infections play an important role in OM. Since most of the MEF samples correspond to OME cases (42 of 49), of which at least 15 are chronic, this is in accordance with the disease continuum hypothesis. According to this theory, *S. pneumoniae* is often involved in an initial AOM episode, which causes mucosal damage and predisposes children to subsequent more complex episodes of RAOM and COME. These most frequently involve non-typeable *H. influenzae* alone or a mixed-species infection that can be present in the form of biofilms [44]. 

NP colonization is a usual first step in OM development [5]; therefore, a correlation between species present in the NP and MEF was already expected. However, recent studies suggest that the NP reservoir theory may not explain all etiologies of MEF production [36]. Next-generation sequencing studies have reported similarities [45] and dissimilarities [46] between the MEF and the adenoidal microbiome in children with OME. Therefore, more extensive investigations are needed to validate or disprove the existing theory.

This study presented some limitations. The bioburden could not be quantified with accuracy since the volume of MEF varied between samples, and some of them were diluted in saline during the collection procedure, while others were not. Therefore, qPCR was used only as a qualitative method instead of quantitative. Moreover, using the universal SYBR Green fluorescent dye instead of specific probes for each bacterial pathogen could have reduced the specificity of the qPCR reactions. Furthermore, the presence of other pathogens, such as *A. otitis*, which may play an important role in both AOM and OME [47,48], was not assessed. Indeed, novel species, particularly *Alloiococcus* and *Turicella*, are being identified at significant relative abundances in MEF samples using metagenomic DNA sequencing, which captures all available DNA in a non-discriminatory manner [36].

## 5. Conclusions

To our knowledge, this is the first study investigating the prevalence of bacterial pathogens in the middle ear fluid of children with otitis media undergoing myringotomy in Portugal. The majority of the cases had a known diagnosis corresponding to otitis media with effusion, with *H. influenzae* being the most prevalent causative agent. qPCR was substantially more sensitive than culturing to detect this pathogen, showing the importance of using molecular methods. However, this was not valid for *S. pneumoniae* and *M. catarrhalis*, which were more frequently detected by culture. The development of *H. influenzae* vaccines, which currently only target *H. influenzae* type B, will be important to reduce the burden of the disease. Moreover, a better understanding of the prevalence of these pathogens and others not included in this study could be achieved by using next-generation sequencing methods, which would give information about the whole middle ear microbiome.

## Figures and Tables

**Table 1:** Bacterial strains and growth conditions.

Species	Strain	Origin or Source	Growth Conditions	Culture Media
*S. pneumoniae*	R6st	Félix d’Hérelle Reference Center for Bacterial Viruses	37 °C, 5% CO_2_	Todd Hewitt Broth + 2% (*w*/*v*) yeast extract
*H. influenzae*	C894248	Sputum, Hospital de Braga	37 °C, 5% CO_2_	Brain Heart Infusion Broth + 10 µg/mL NAD + 10 µg/mL Hemin
*M. catarrhalis*	U225012	Ocular, Hospital de Braga	37 °C, 5% CO_2_	Brain Heart Infusion Broth
*S. aureus*	ATCC 6358	Human lesion, American Type Culture Collection	37 °C	Tryptic Soy Broth
*P. aeruginosa*	PAO1 (DSM 22644)	Infected wound, DSMZ—German Collection of Microorganisms and Cell Cultures GmbH	37 °C	Tryptic Soy Broth

**Table 2:** List of primers for specific PCR detection of target pathogens.

Target	Primer Name	Primer Sequence (5′ → 3′)	Product Size
*S. pneumoniae*, *oralis*, *mitis* and *infantis* 16S rRNA gene	Spomi_FW	AAGGTGCACTTGCATCACTACC	484
	Common_RV	CTACGCATTTCACCGCTACAC	
*H. influenzae* 16S rRNA gene	Hi_FW	CGTATTATCGGAAGATGAAAGTGC	525
	Common_RV	CTACGCATTTCACCGCTACAC	
*M. catarrhalis* 16S rRNA gene	Mc_FW	CCCATAAGCCCTGACGTTAC	237
	Common_RV	CTACGCATTTCACCGCTACAC	
*S. pneumoniae* lytA gene	Sp_FW	ACGCAATCTAGCAGATGAAGCA	76
	Sp_RV	TCGTGCGTTTTAATTCCAGCT	
*S. aureus* nucA gene	Sa_FW	CATCCTAAAAAAGGTGTAGAGA	85
	Sa_RV	TTCAATTTTMTTTGCATTTTCTACCA	
*P. aeruginosa* oprL gene	Pa_FW	GGGTTCATTAGGAGTTACATGA	544
	Pa_RV	GGGCATAACGACTTCTTACTTC	

## Data Availability

Not applicable.

## Supplementary Materials

The following are available online at https://www.mdpi.com/article/10.3390/microorganisms10010054/s1, Figure S1: Agarose gel electrophoresis of amplified genes, Figure S2: Standard curves and melting curves for qPCR quantification of the internal exogenous control (pET-28a(+)), Figure S3: Standard curves and melting curves for qPCR quantification of middle ear fluid bacterial pathogens, Table S1: Demographic information (age, sex) and clinical characteristics of the study subjects.
