# Supplementary material for: Identification of the Bacterial Pathogens in Children with Otitis Media: A Study in the Northwestern Portuguese District of Braga
Source: Microorganisms. 2021 Dec 27;10(1):54. doi: 10.3390/microorganisms10010054 (PMC8779683; doi:10.3390/microorganisms10010054)
Supplement: Supplementary file 1 [file microorganisms-10-00054-s001.zip › microorganisms-1517836-supplementary.pdf]

Supplementary material

Bacterial species present in children with otitis media in the northwestern Portuguese district of Braga

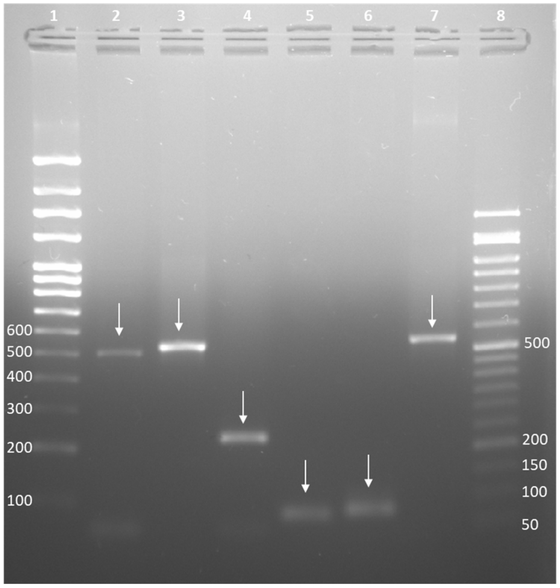

Figure S1. Agarose gel electrophoresis (2%) of amplified genes (1: NZYDNA Ladder VII; 2: *S. pneumoniae* 16S rRNA; 3: *H. influenzae* 16S rRNA; 4: *M. catarrhalis* 16S rRNA; 5: *S. pneumoniae lytA*; 6: *S. aureus nucA*; 7: *P. aeruginosa oprL*; 8: NZYDNA Ladder VI).

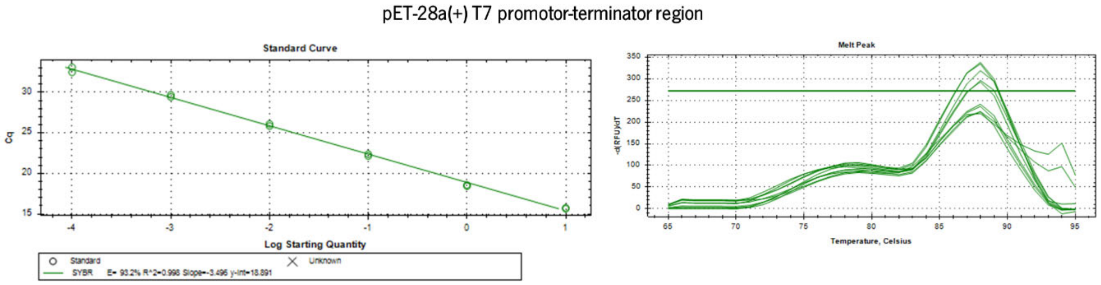

Figure S2. Standard curves (left) and melting curves (right) for qPCR quantification of the internal exogenous control (pET-28a(+)).

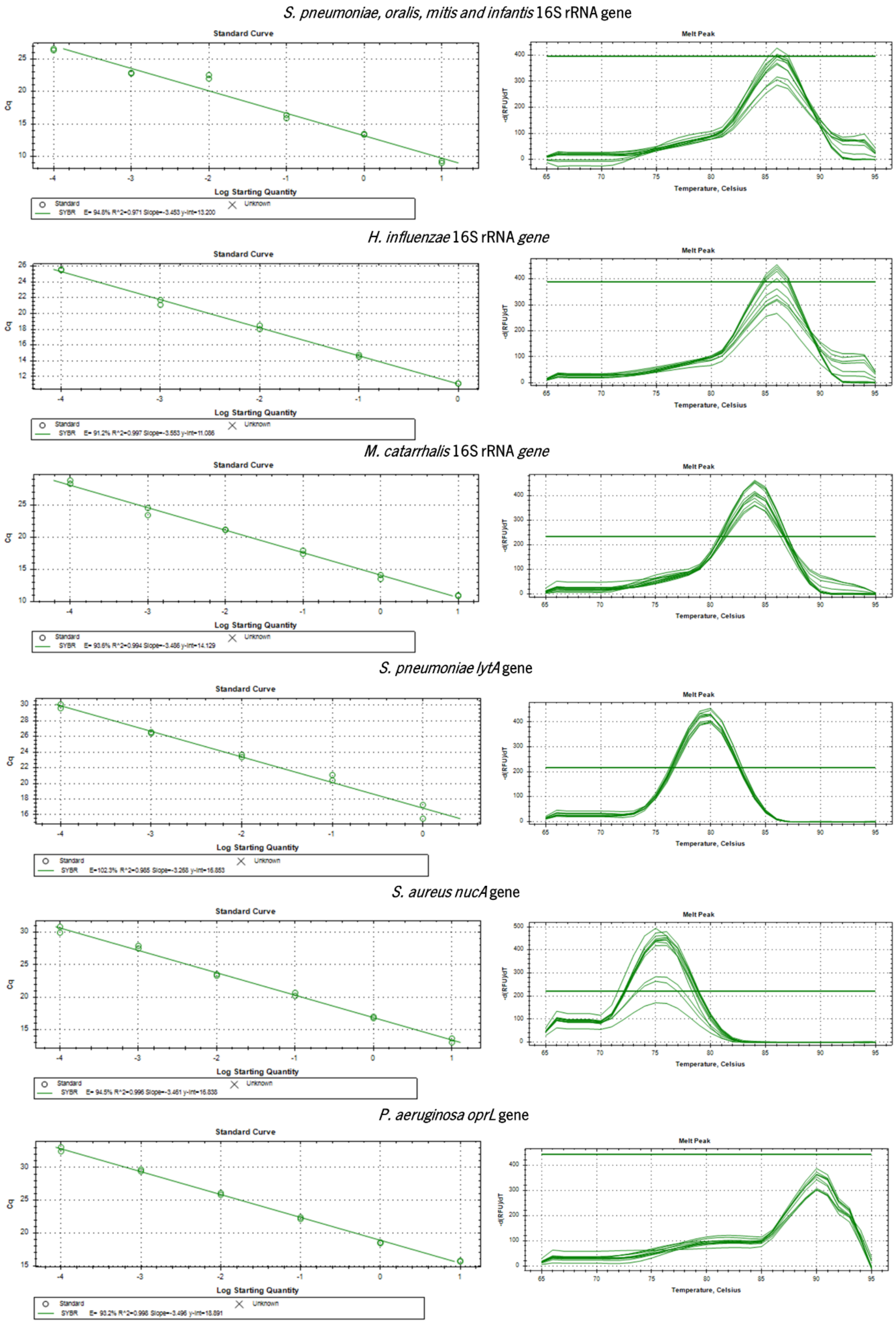

Figure S3. Standard curves (left) and melting curves (right) for qPCR quantification of middle ear fluid bacterial pathogens.

Table S1. Demographic information (age, sex) and clinical characteristics of the study subjects

Patient	Age	Sex	Type of OM	Lesion	Myringotomy	Adenoidectomy	Antibiotics	Pneumococcal vaccine
**1**	9	M	OME	Right	Reintervention	Yes	NA	No
**2**	5	F	RAOM	Bilateral	Reintervention	Yes	AMC	Yes
**3**	5	F	OME	Left	First	Yes	NA	Yes
**4**	6	F	OME	Left	First	Yes	AMC	Yes
**5**	2	F	OME	Bilateral	First	Yes	NA	No
**6**	4	F	OME	Bilateral	First	Yes	AMC	No
**7**	2	M	OME	Right	First	Yes	PEN G	Yes
**8**	2	M	OME	Right	First	No	NA	Yes
**9**	3	M	OME	Right	First	Yes	AMC	Yes
**10**	5	M	OME	Right	First	Yes	AMC	Yes
**11**	3	F	OME	Right	First	Yes	AMC	Yes
**12**	3	F	OME	Right	First	Yes	NA	Yes
**13**	5	M	OME	Bilateral	First	Yes	NA	Yes
**14**	4	F	OME	Bilateral	First	Yes	NA	Yes
**15**	2	M	OME	Right	First	Yes	AMC	Yes
**16**	2	M	OME	Right	First	Yes	PEN	Yes
**17**	2	M	OME	Bilateral	First	Yes	AMC	Yes
**18**	3	F	OME	Bilateral	First	Yes	NA	Yes
**19**	1	F	OME	Bilateral	First	Yes	NA	Yes
**20**	8	M	OME	Left	First	Yes	AMC	Yes
**21**	7	F	OME	Bilateral	First	Yes	NA	Yes
**22**	3	M	OME	Right	First	Yes	AMC	Yes
**23**	1	F	OME	Bilateral	First	Yes	NA	Yes
**24**	1	F	RAOM	Bilateral	First	No	CRO	Yes
**25**	3	M	OME	Right	First	Yes	AMC	Yes
**26**	3	M	OME	Bilateral	First	Yes	AMC	Yes
**27**	5	F	OME	Left	First	Yes	NA	Yes
**28**	3	F	OME	Left	First	Yes	AMC	Yes
**29**	2	F	OME	Bilateral	First	Yes	NA	Yes
**30**	3	M	OME	Right	First	Yes	AMC	Yes
**31**	4	F	OME	Left	First	Yes	NA	Yes
**32**	3	M	OME	Bilateral	First	Yes	AMC	Yes
**33**	3	F	OME	Right	First	Yes	NA	Yes
**34**	2	F	OME	Bilateral	First	Yes	CFM	Yes
**35**	3	M	OME	Bilateral	First	Yes	NA	Yes
**36**	2	M	OME	Right	First	Yes	CFM	Yes
**37**	4	M	OME	Right	First	Yes	NA	Yes
**38**	4	F	OME	Left	First	Yes	AMC	Yes

**39**	4	M	OME	Right	First	Yes	NA	Yes
**40**	4	F	OME	Bilateral	First	Yes	AMC	Yes
**41**	2	M	OME	Bilateral	First	Yes	NA	Yes
**42**	4	F	OME	Left	First	Yes	NA	Yes
**43**	2	M	OME	Bilateral	First	Yes	AMC	Yes
**44**	2	M	OME	Bilateral	First	Yes	NA	Yes
**45**	4	M	OME	Right	First	Yes	AMC	Yes
**46**	4	M	OME	Left	First	Yes	NA	Yes
**47**	4	F	OME	Left	First	Yes	NA	Yes
**48**	4	M	OME	Bilateral	First	Yes	AMC	Yes
**49**	4	F	OME	Right	First	Yes	AMC	Yes

M: male; F: female; RAOM: recurrent acute otitis media; OME: otitis media with effusion; NA: not administered; AMC: amoxicillin/clavulanic acid; PEN: penicillin; CRO: ceftriaxone; CFM: cefixime
